# Supplementary material for: Protective anti‐prion antibodies in human immunoglobulin repertoires
Source: EMBO Mol Med. 2020 Aug 10;12(9):e12739. doi: 10.15252/emmm.202012739 (PMC7506995; doi:10.15252/emmm.202012739)
Supplement: Supplementary file 1 — Appendix [file EMMM-12-e12739-s001.pdf]

Appendix information

Table of content:

1. **Appendix Figure S1**
2. **Appendix Figure S2**
3. **Appendix Figure S3**
4. **Appendix Figure S4**
5. **Appendix Figure legends**
6. **Appendix Table S1**
7. **Appendix Table S2**
8. **Appendix Table S3**
9. **Appendix Table S4**
10. **Appendix Table S5**
11. **Appendix Table S6 (exact p-values)**

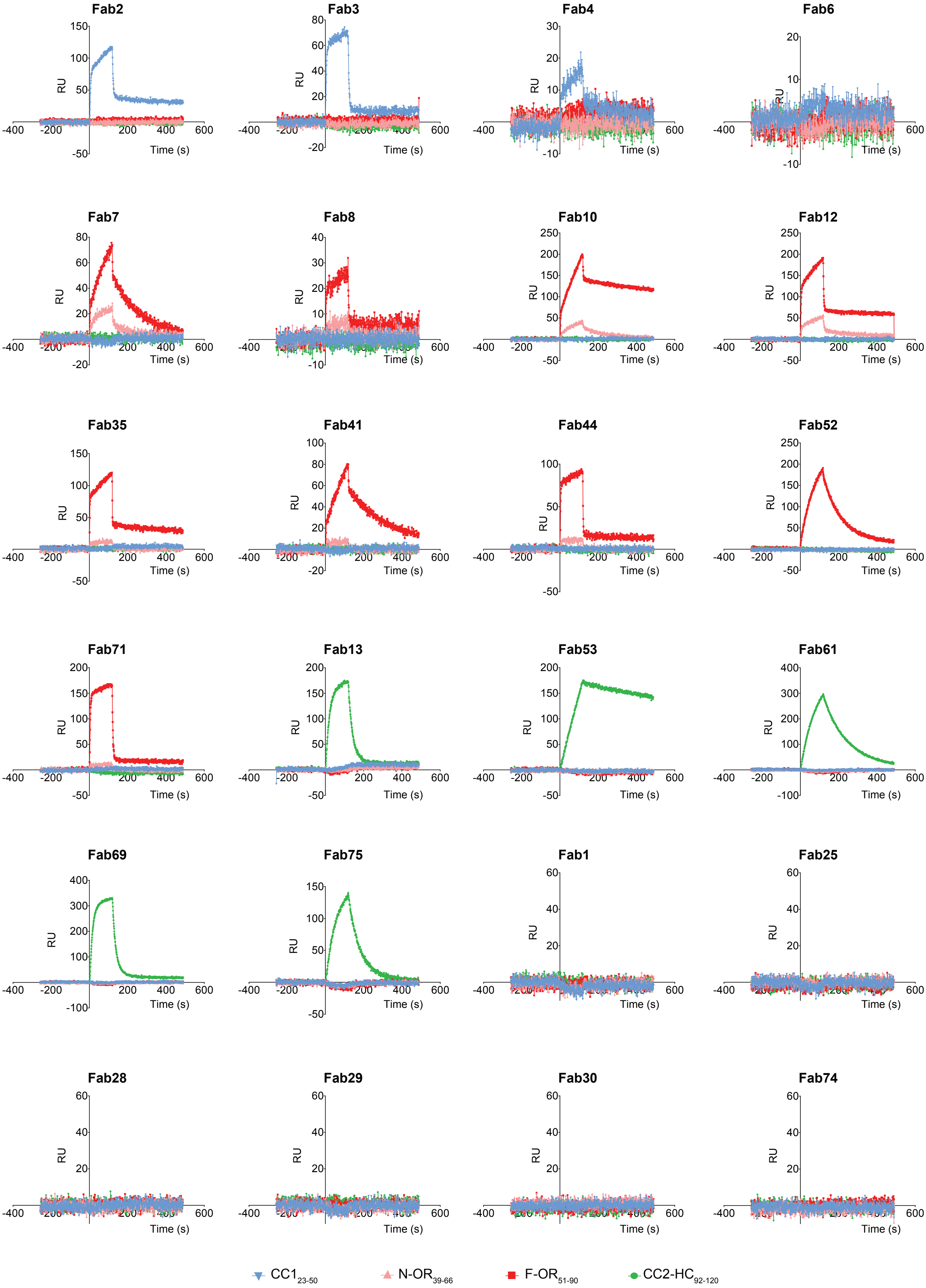

Senatore et al., Appendix Figure S1

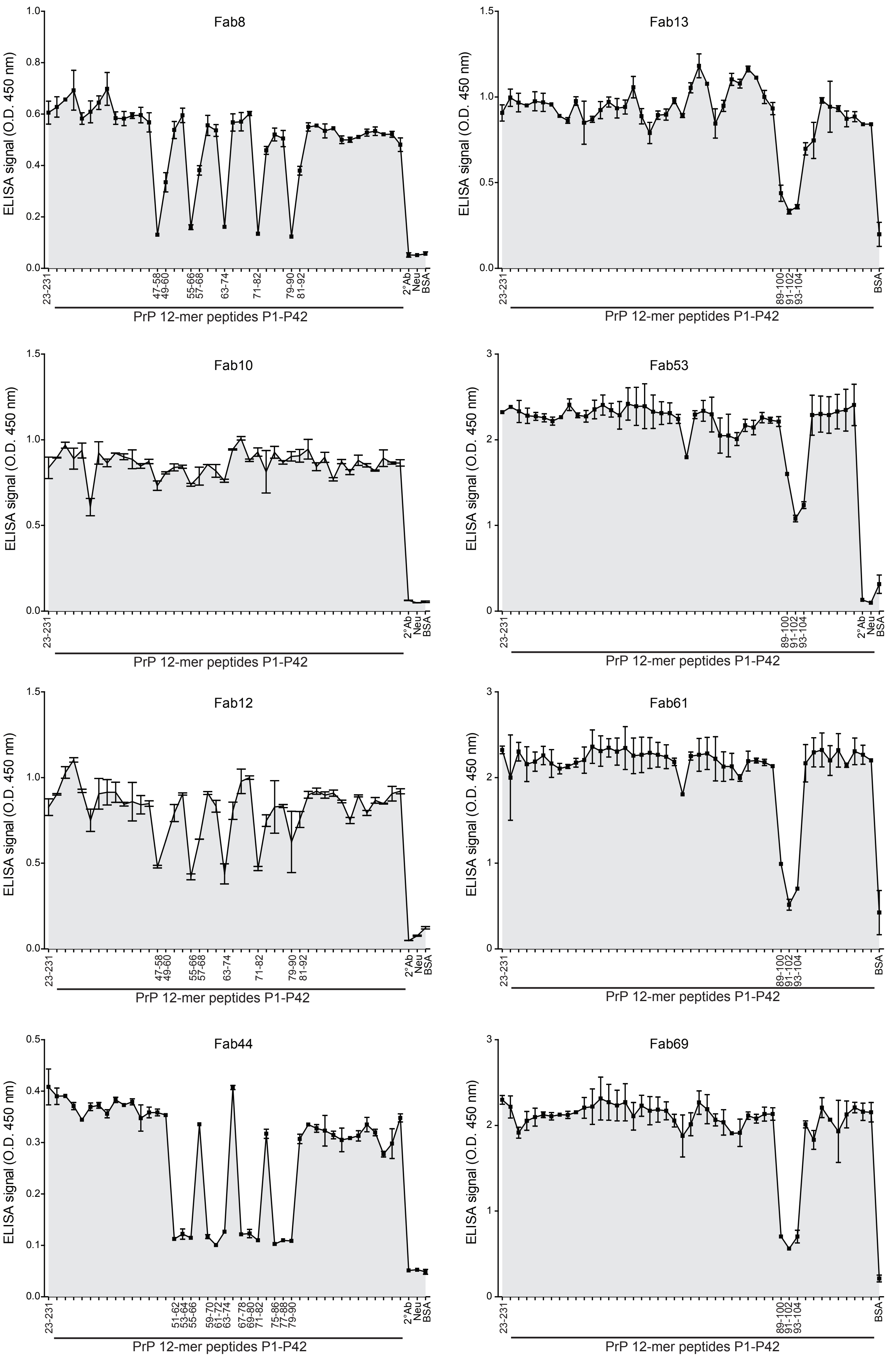

Senatore et al., Appendix Figure S2

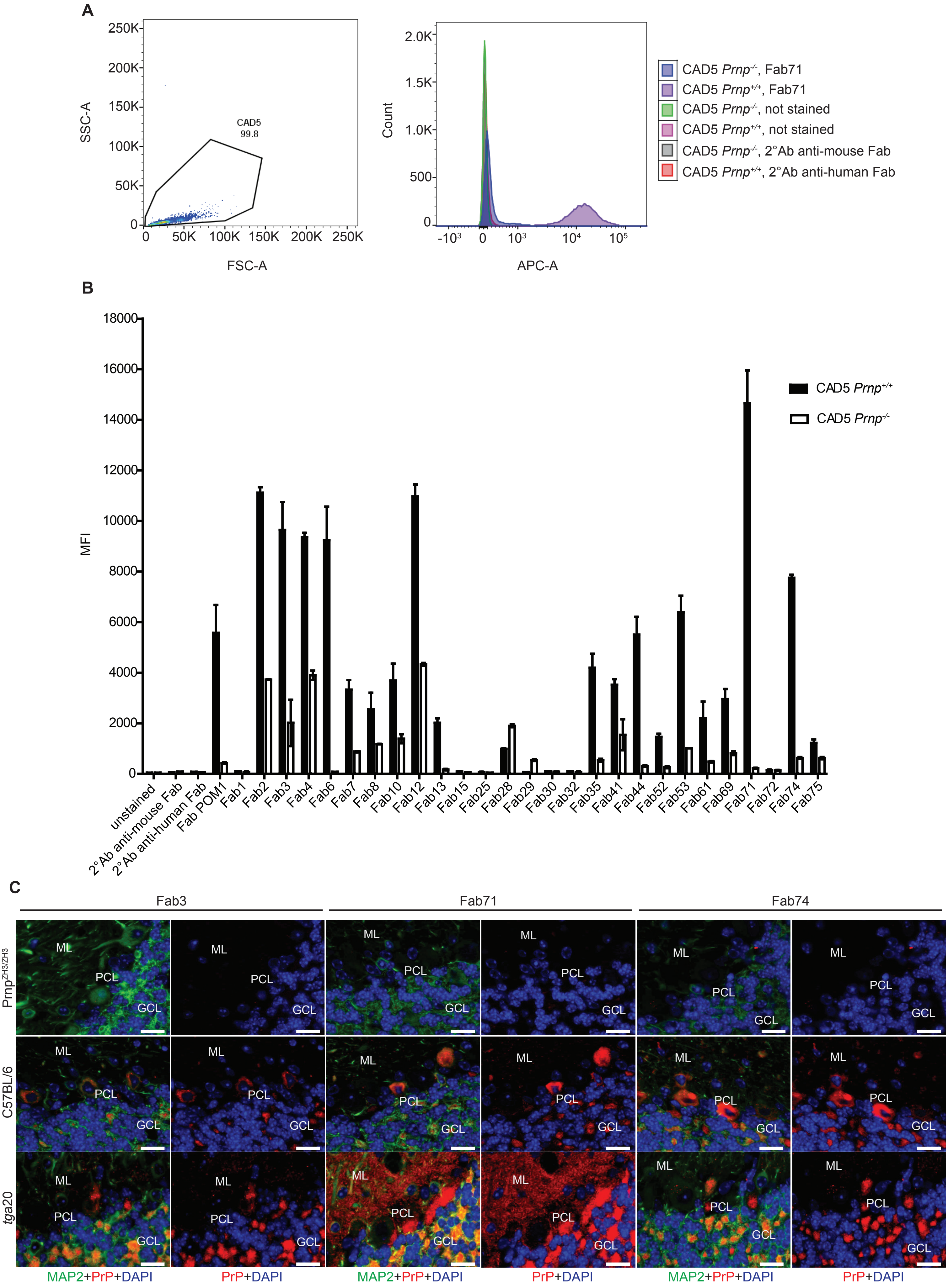

Senatore et al., Appendix Figure S3

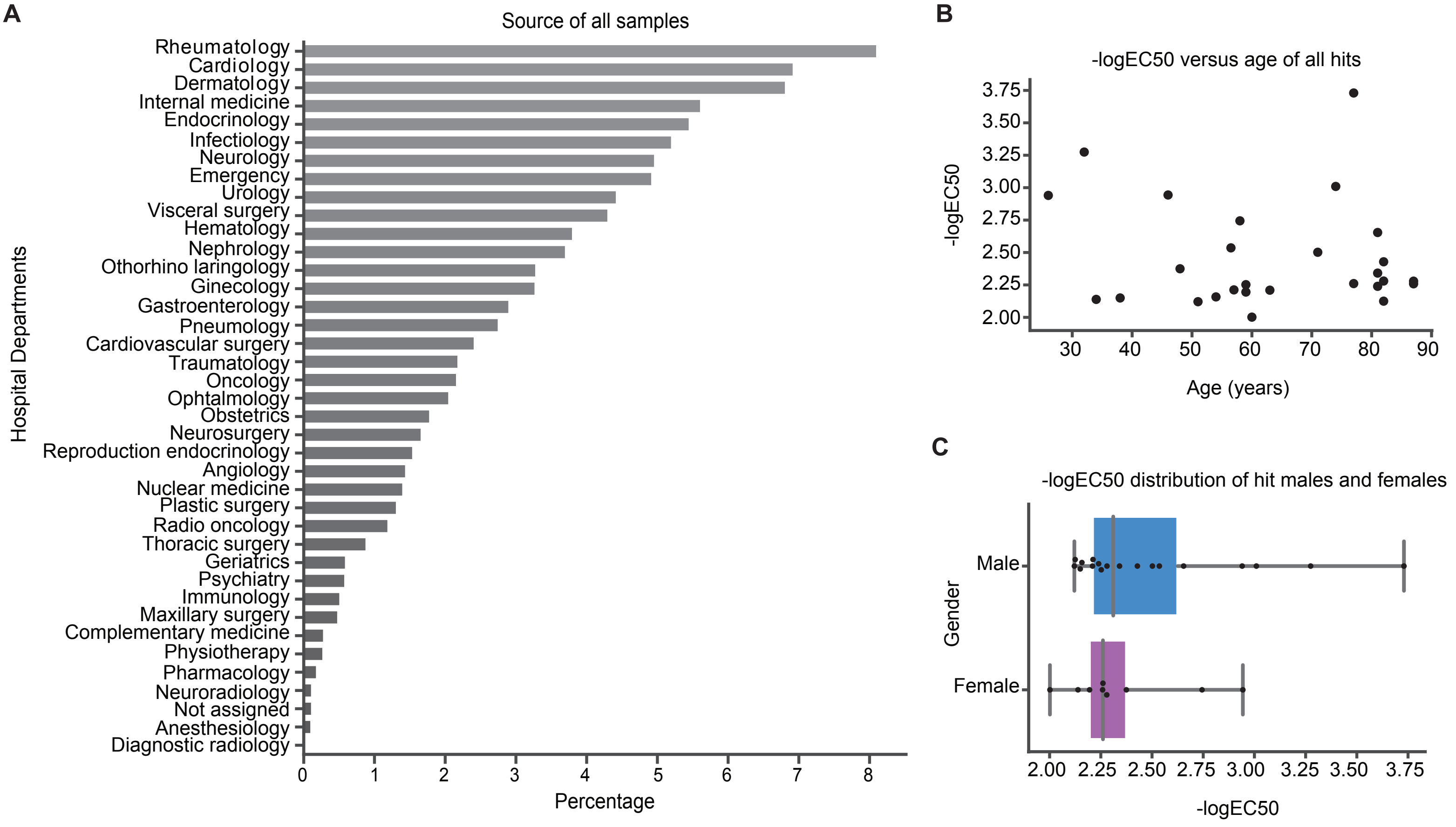

Senatore et al., Appendix Figure S4

Appendix figure legends

Appendix Fig. S1: Epitope confirmation by SPR. Biotinylated PrP peptides were immobilized on an NLC sensor chip and the binding of the indicated Fabs was measured by SPR. Epitope assessment by SPR confirmed the results obtained by ELISA. RU: relative units.

Appendix Fig. S2: Epitope mapping by competition ELISA. FT-competition ELISA to map the epitopes of the OR_{51-91} binders (Fab8, Fab10, Fab44, Fab12) and $CC2\text{-}HC_{92-120}$ binders (Fab13, Fab53, Fab61, Fab69). Peptides that strongly inhibit the binding of the Fabs to $recPrP_{23-231}$ are indicated by their residue numbers in the PrP sequence and reflect the respective binding epitopes. Positive control: $recPrP_{23-231}$. Negative controls: Neu, BSA, and 2° Ab.

Data information: ELISA data were performed in duplicates. Data represent the mean ± sem.

Appendix Fig. S3: Anti-PrP Fabs detect cell surface exposed $wtPrP^C$ and stain $wtPrP^C$ in paraffin embedded mouse brain sections.

(A) Flow cytometry analysis of the Fabs compared to Fab-POM1 used as positive control for the staining. CAD5 *Prnp*$^{+/+}$ and *Prnp*$^{-/-}$ cells were incubated with the Fabs at 150 µg/ml, and analyzed with an APC-labelled anti-human Fab antibody. Representative flow cytometry dot plot of FSC-A (forward scatter)/SSC-A (side scatter) of CAD5 *Prnp*$^{+/+}$ stained with Fab71 (left) and fluorescence intensity plot of CAD5 *Prnp*$^{+/+}$ and *Prnp*$^{-/-}$ cells.

(B) Bar graph showing the average mean fluorescent intensity (MFI) for each Fab. Increased MFIs indicate binding of the Fabs to $wtPrP^C$ on the cell surface. Each Fab was tested in duplicate.

Data information: Data represent the mean ± sem.

(C) Immunofluorescent staining of cerebellar brain sections of *Prnp*$^{ZH3/ZH3}$, wt and *tg*a20 mice with either Fab3, Fab71, or Fab74 (displayed in red). Anti-MAP2 antibody (microtubule-associated protein 2) was used as neuronal marker (in green) and DAPI to stain the cell nuclei

(in blue). The Fabs detected wtPrP^{C} in the cerebellar granule cell layer (CGL) and molecular layer (ML) of wt and *tg*a20 mice. As expected, the Fabs did not detect wtPrP^{C} in Purkinje cell layer (PCL) of *tg*a20 mice. The higher staining intensity of Fab71 might be caused by its ability to recognize the repetitive epitopes within the OR multiple times. Scale bar: 20 µm.

Appendix Fig. S4: Medical and demographic data of the hospital cohort screened for anti-PrP antibodies and of the hits identified in the primary screening.

(A) Bar plot indicating the hospital departments from which the tested 37,894 samples were obtained.

(B,C) Correlations between -log(EC50) and age (B), -log(EC50) and sex (C). Black dots in panels (B) and (C) indicate each of the 27 hits that were identified in the primary screen.

Data information: The boxplot divides the data set into three quartiles. The box extends from the first to the third quartile values of the data set, with a line at the median. The whiskers extend from the box to show the range of the data (minimum and maximum).

Appendix Table S1: Matrix of the different washing conditions during panning

Washing time	A1 (standard)	A2 (high salt)	A3 (detergent)
5 times quick, 10 times 5 min	PBST	PBST + 1M NaCl	PBS + 2% Octyl β-D-glucopyranoside
5 times quick, 7 times 5 min	PBS	PBS + 1M NaCl	PBS + 2% Octyl β-D-glucopyranoside
3 times 5 min	PBS	PBS	PBS

Appendix Table S2: Criteria for sorting the NGS counts for epitope prediction of the anti-PrP Fabs

**Panning output NGS libraries**															
			**Ref**	**binding strength**		**synthetic biotinylated PrP peptides**					**recombinant PrP fragments**			**negative controls**	
**mouse PrP amino acid residues**	**PrP domains**		**recPrP$_{23\text{-}231}$**	**recPrP$_{23\text{-}231}$ high salt**	**recPrP$_{23\text{-}231}$ detergent**	**CC1 $_{23\text{-}50}$ SP**	**CC1 $_{23\text{-}50}$ LP**	**N-OR $_{39\text{-}66}$**	**F-OR $_{51\text{-}91}$**	**CC2-HC $_{92\text{-}120}$**	**recPrP$_{23\text{-}110}$**	**recPrP$_{90\text{-}231}$**	**recPrP$_{121\text{-}231}$**	**Neutravidin**	**BSA**
**23-38**	**CC1**	**CC1_SP**	1	ns	ns	>0	ns	0	0	0	>0	0	0	0	0
		**CC1_LP**	1	ns	ns	ns	>0	0	0	0	>0	0	0	0	0
**39-50**	**CC1-N-OR**		1	ns	ns	ns	ns	>0	0	0	>0	0	0	0	0
**50-66**	**N-OR**		1	ns	ns	0	0	>0	ns	0	>0	0	0	0	0
**51-91**	**F-OR**		1	ns	ns	0	0	ns	>0	0	>0	0	0	0	0
**92-110**	**CC2-HC**		1	ns	ns	0	0	0	0	>0	>0	>0	0	0	0
**111-120**	**CC2-HC**		1	ns	ns	0	0	0	0	>0	0	>0	0	0	0
**90-231**	**CC2-GD**		1	ns	ns	0	0	0	0	0	0	>0	>0	0	0
**121-231**	**GD**		1	ns	ns	0	0	0	0	0	0	0	>0	0	0

Ref: reference panning to recPrP$_{23\text{-}231}$

CC1_SP: CC1 Solid Phase

CC1_LP: CC1 Liquid Phase

ns: not selected

Appendix Table S3: EC50 of Fab3 and Fab71 and their respective affinity matured variants as determined by ELISA

	**EC_{50} (nM)**	**R square**
**Fab3**	30.430	0.965
**Fab81**	0.184	0.987
**Fab82**	0.298	0.984
**Fab83**	0.044	0.982
**Fab84**	0.530	0.990
**Fab85**	0.465	0.989
**Fab86**	0.541	0.981
**Fab87**	0.385	0.991
**Fab88**	0.474	0.989
**Fab89**	0.421	0.993
**Fab90**	0.235	0.961
**Fab91**	0.450	0.984
**Fab92**	0.260	0.992
**Fab93**	0.518	0.989
**Fab94**	0.210	0.992
**Fab95**	0.511	0.994
**Fab96**	1.516	0.987
**Fab97**	0.322	0.977
**Fab98**	1.005	0.990
Fab99	1.119	0.995

	**EC_{50} (nM)**	**R square**
**Fab71**	24.890	0.992
**Fab100**	0.232	0.983
**Fab101**	0.190	0.964
**Fab102**	0.238	0.981
**Fab103**	0.266	0.976
**Fab104**	0.380	0.958

Appendix Table S4: **Features and clinical data of the subjects displaying high titre of anti-PrP autoantibodies in the plasma.**

Patient ID	Core diagnoses	Sex	Year of birth
**Patient_01**	Valvular and coronary heart disease; chronic obstructive pulmonary disease	Male	1943
**Patient_02**	Contusion of digit	Female	1971
**Patient_03**	Chronic lymphatic leukaemia; valvular cardiac disease; chronic kidney insufficiency; oncocytoma	Male	1937
**Patient_04**	Not shown		
**Patient_05**	Myeloid leukaemia	Male	1959
**Patient_06**	Aortic stenosis	Male	1947
**Patient_07**	Not shown		
**Patient_08**	Aortic aneurysm and dissection	Male	1937
**Patient_09**	Ulcerating colitis; drug abuse	Female	1970
**Patient_010**	Not shown		
**Patient_014**	HIV infection; erectile dysfunction; gonarthrosis	Male	1959
**Patient_015**	Intraspinal space-consuming lesion (ganglioneurom WHO grade I); drug abuse	Male	1985
**Patient_016**	Coronary heart disease; severe kidney insufficiency; hypothyreosis	Female	1959
**Patient_017**	Polyserositis; mamma carcinoma	Female	1958
**Patient_018**	HIV infection	Male	1991
**Patient_019**	Not shown		
**Patient_020**	Urothel carcinoma; adenocarninoma; arrythmogenic cardiac disease	Male	1936
**Patient_021**	Coronary 2-vessel disease	Male	1954
**Patient_022**	Coronary heart disease; neuropathic pain	Male	1935
**Patient_023**	T cell lymphoma	Male	1967
**Patient_024**	Injury of the pelvic ring	Male	1964
**Patient_025**	Status epilepticus; myxofibrosarcoma; slight dementia type Alzheimer's disease	Female	1931
**Patient_026**	Squamos cell carcinoma	Female	1941
**Patient_027**	Not shown		
**Patient_028**	Syncope; severe depressive episodes	Female	1985
**Patient_029**	Not shown		
**Patient_030**	Adenocarcinoma; signs of dementia	Male	1937

Shown are the core diagnoses, the sex, and the year of birth for the 21 out of 27 individuals identified with autoantibodies against recombinant PrP in the high-throughput antibody profiling. We have refrained from displaying any patient-associated information from patients who do not gave expressive consent by signing the hospital-wide general consent of the University Hospital of Zurich. While many but not all of the patients included here are highly multimorbid, only the core diagnoses were selected for display, also with respect to the appropriate handling of sensitive patient data.

Appendix Table S5: Comparison of age, gender and clinical data between subjects with and without high titre PrP autoantibodies.

Median age hits	# hits	Median age non-hits	# non-hits	P-value
61	27	55	48691	3.58E-02

% male hits	% female hits	% male non-hits	% female non-hits	P-value
66.67	33.33	52.42	47.64	1.96E-01

ICD-10 code	# hits	# non-hits	# hits	# non-hits	P-value	Bonferroni
E87	6	4830	22.22	9.92	4.56E-02	1.82E-01
I25	7	6207	25.93	12.75	7.33E-02	2.93E-01
I10	9	11595	33.33	23.81	2.59E-01	1
N18	5	5874	18.52	12.06	3.76E-01	1

Statistical testing for difference in age (median test, significance at alpha ≤ 0.01) or gender (chi-square test, significance at alpha ≤ 0.01) revealed no significant difference. Analysis of enriched ICD-10 codes in patients with anti-PrP-autoantibodies versus patients without. No patterns could be discerned and no ICD-10 code reached a statistically significant enrichment (chi-square test, significance at alpha ≤ 0.01, Bonferroni correction for multiple comparisons).

Appendix Table S6: exact *p*-values

**Figure panel**	**comparison**	***p*-value**
**Figure 3C**	NBH: untreated vs Fab 25	0.000683
	Prion: untreated vs Fab83	0.001199
	Prion: untreated vs Fab8	3.6E-14
	Prion: untreated vs Fab44	6.67E-12
	Prion: untreated vs Fab71	9.89E-10
	Prion: untreated vs Fab100	7.16E-10
**Figure 3D**	untreated vs Fab100	0.003309
**Figure EV3C**	CAD5-Prnp-/- non transfected vs WtPrPC	0.0001
	CAD5-Prnp-/- non transfected vs PrPΔ23-31	0.006
	CAD5-Prnp-/- non transfected vs PrPΔ23-27	0.0148
	CAD5-Prnp-/- non transfected vs 〖PrP〗_(23-27)^(K→A)	0.0001
	CAD5-Prnp-/- non transfected vs 〖PrP〗_(23-27)^Mirror	0.0025
**Figure EV5A**	Prion vs Prion + Fab71	0.001173
**Figure EV5B**	Prion vs Prion + Fab100	0.019613
