# Supplementary material for: Protective anti‐prion antibodies in human immunoglobulin repertoires
Source: EMBO Mol Med. 2020 Aug 10;12(9):e12739. doi: 10.15252/emmm.202012739 (PMC7506995; doi:10.15252/emmm.202012739)
Supplement: Supplementary file 2 — Expanded View Figures PDF [file EMMM-12-e12739-s002.pdf]

Expanded View Figures

Figure EV1. Selection strategy to identify anti-PrP Fabs from a synthetic human Fab phage library and rescue of NGS-identified Fab clones.

A RecPrP$_{23\text{-}231}$ (blue boxes) was used as target for the first and second round of panning. In the third round, selected phages were panned against recPrP$_{23\text{-}231}$, recPrP$_{23\text{-}231}$ under different washing conditions, recPrP fragments (red boxes) and peptides (green boxes) to select for Fabs targeting specific regions of PrP. The DNA preparation from the selected phages was used for NGS of the HCDR3 or ELISA. Negative controls: BSA and Neu (gray boxes).

B Bar plot of the NGS counts, relative to recPrP$_{23\text{-}231}$, for the indicated HCDR3 sequence.

C PCR strategy to retrieve the clone of interest. Specific HCDR3_Rw and HCDR3_Fw primers (red arrows) were designed as complementary to the sequence to be rescued. In two separate PCR reactions, the HCDR3_Rw primer was used in combination with the Pal_Fw primer (blue arrow) annealing upstream of the VL, whereas the HCDR3_Fw primer was used in combination with the Flag_RW primer annealing to the His-tag sequence (blue arrow). The two amplicons were then assembled in a second PCR reaction resulting in the full Fab sequence.

D The retrieved Fab clone (FabRTV) was expressed in *E. coli* and purified by IMAC. The purity of the Fab was analyzed by SDS–PAGE.

E FabRTV was tested for its binding specificity to recPrP$_{23\text{-}231}$ and the indicated PrP fragments and peptides by ELISA. As determined by the NGS analysis (B) and given as an example, FabRTV binds to CC2-HC$_{92\text{-}120}$.

Data information: ELISA data were performed in duplicates. Data represent the mean ± sem.

▶

© 2020 The Authors

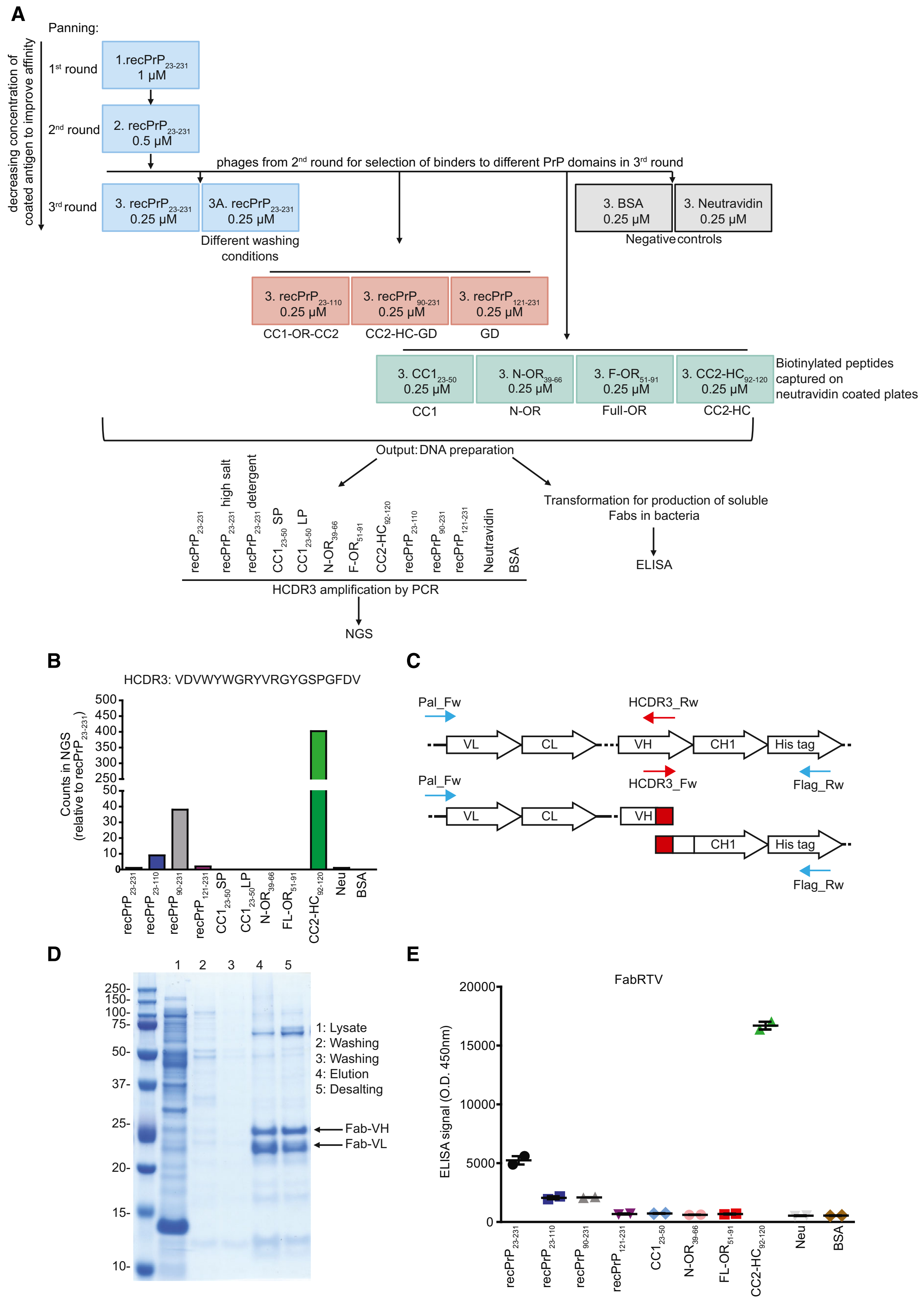
A
Panning:
decreasing concentration of coated antigen to improve affinity
1st round
1.recPrP23-231 1 µM
2nd round
2. recPrP23-231 0.5 µM
phages from 2nd round for selection of binders to different PrP domains in 3rd round
3rd round
3. recPrP23-231 0.25 µM
3A. recPrP23-231 0.25 µM
Different washing conditions
3. BSA 0.25 µM
3. Neutravidin 0.25 µM
Negative controls
3. recPrP23-110 0.25 µM
3. recPrP90-231 0.25 µM
3. recPrP121-231 0.25 µM
CC1-OR-CC2
CC2-HC-GD
GD
3. CC1 23-50 0.25 µM
3. N-OR39-66 0.25 µM
3. F-OR51-91 0.25 µM
3. CC2-HC92-120 0.25 µM
Biotinylated peptides captured on neutravidin coated plates
CC1
N-OR
Full-OR
CC2-HC
Output: DNA preparation
Transformation for production of soluble Fabs in bacteria
ELISA
HCDR3 amplification by PCR
NGS
B
HCDR3: VDVWYWGRYVRGYGSPGFDV
Counts in NGS (relative to recPrP23-231)
C
Pal_Fw
HCDR3_Rw
VL
CL
VH
CH1
His tag
HCDR3_Fw
Flag_Rw
D
1: Lysate
2: Washing
3: Washing
4: Elution
5: Desalting
Fab-VH
Fab-VL
E
FabRTV
ELISA signal (O.D. 450nm)

Figure EV1.

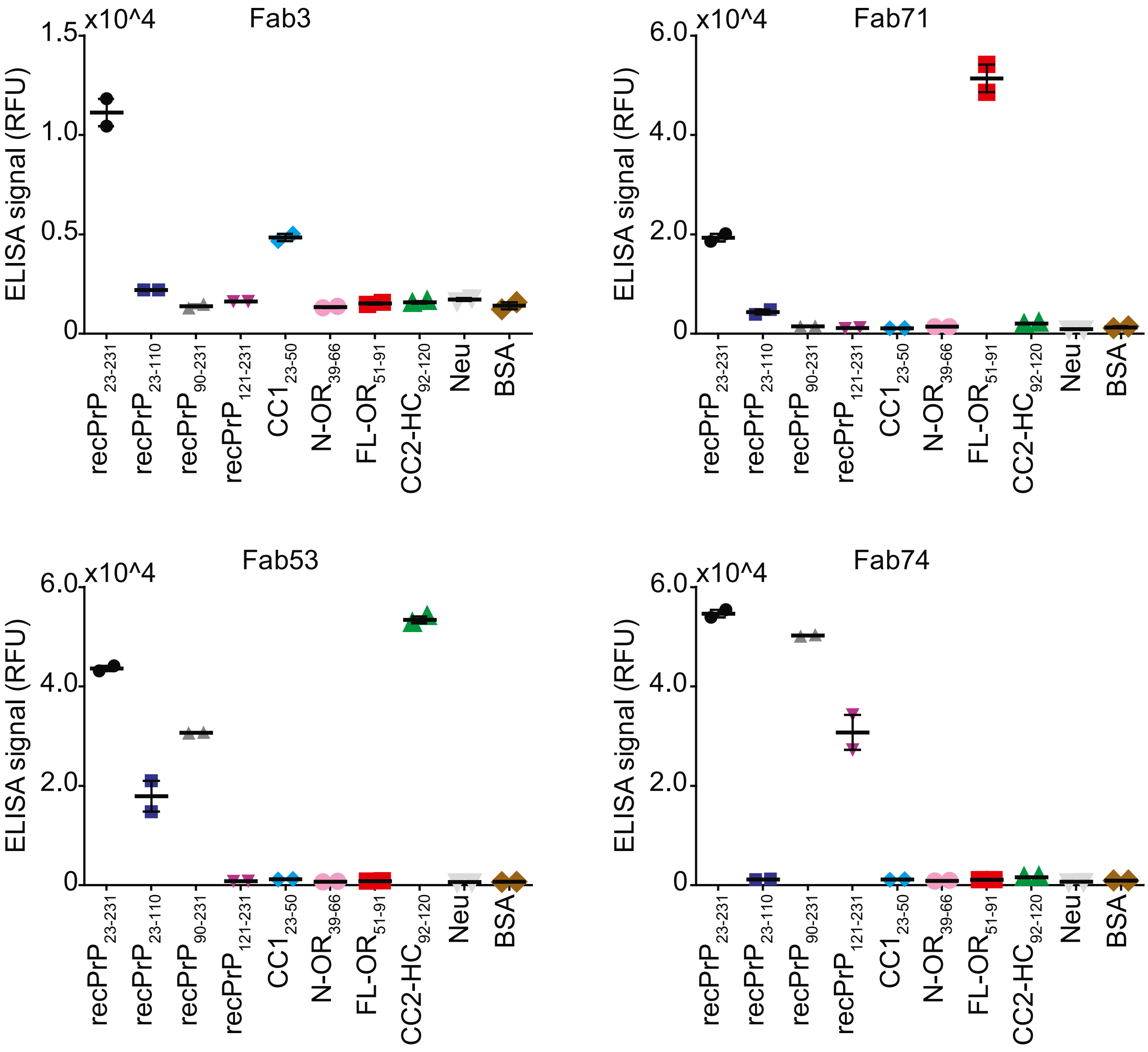

Figure EV2. Binding specificity of the retrieved anti-PrP Fabs.

ELISA signal (relative fluorescence units, RFU) indicating the binding reactivity of selected, purified anti-PrP Fabs to mouse $recPrP_{23-231}$, mouse recPrP fragments and selected N-terminal PrP peptides. Negative controls: Neu and BSA. Fab3: $CC1_{23-50}$ binder; Fab71: OR_{51-91} binder; Fab53: $CC2\text{-}HC_{92-120}$ binder; Fab74: GD binder.
Data information: ELISA data were performed in duplicates. Data represent the mean ± sem.

 © 2020 The Authors

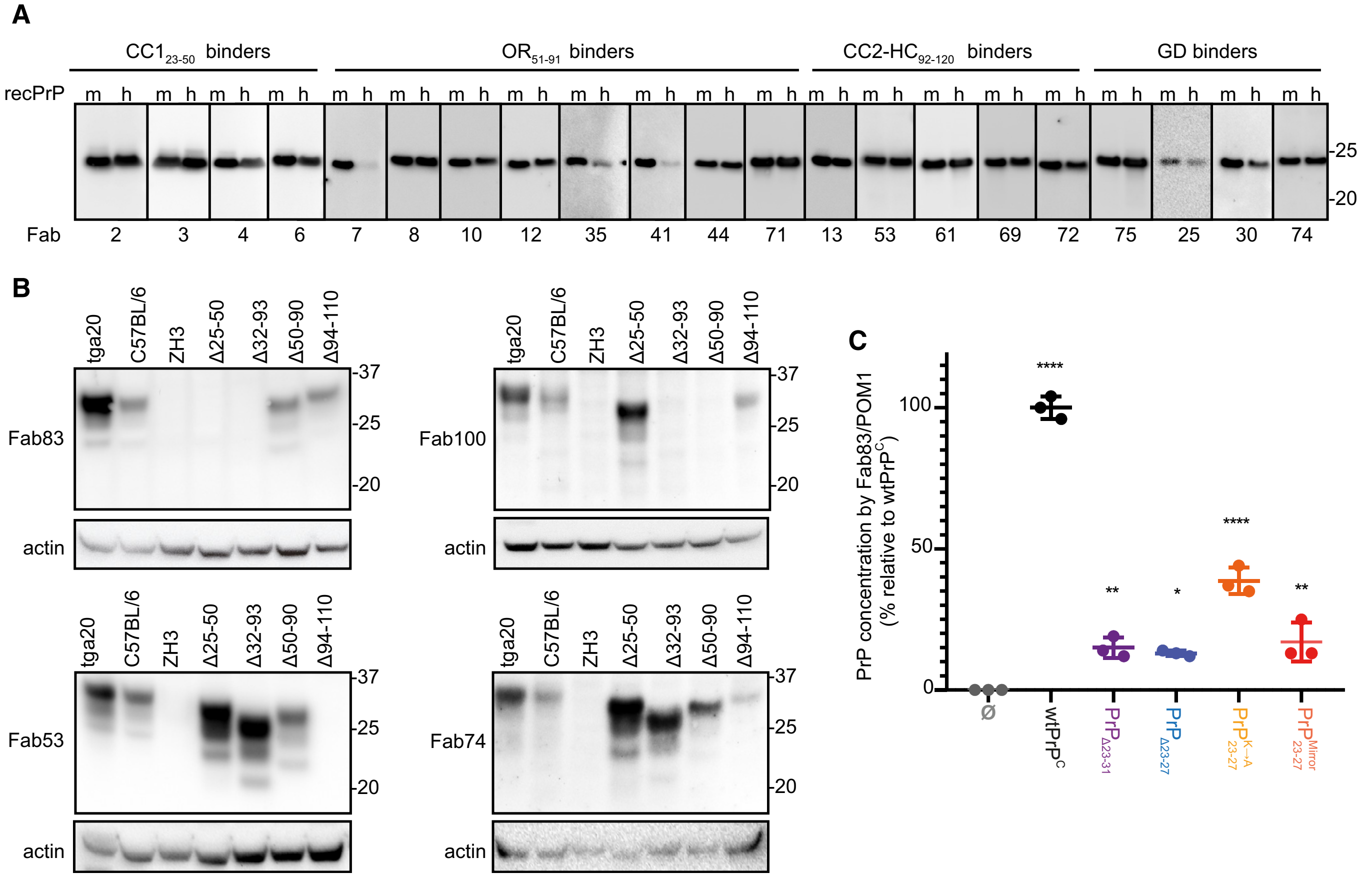

Figure EV3. Validation of the reactivity of the anti-PrP Fabs by Western blot, ELISA, and immunoprecipitation.

A, B Western blot analysis to compare the reactivity of the indicated Fabs to mouse (m) recPrP$_{23\text{-}231}$ and human (h) recPrP$_{23\text{-}230}$ (A) and to full-length and truncated PrP^{C} in BHs of various mouse lines. Actin was used as loading control (B).

C ELISA to compare the efficiency of Fab83 and POM1 to detect and quantify wt and mutant PrP^{C} levels in CAD5-*Prnp*$^{-/-}$ cells transfected either with wt or mutant PrP^{C} (deletion mutants PrPΔ23-31 or PrPΔ23-27; PrP$^{K\rightarrow A}_{23-27}$ with lysine residues 23, 24, and 27 replaced by alanine; PrP$^{Mirror}_{23-27}$ with KKRPK exchanged to KPRKK). All concentrations (% relative to wtPrP^{C}) were determined by interpolating the ELISA signal to a standard curve of mouse recPrP$_{23\text{-}231}$.

Data information: ELISA data were performed in triplicates. Data represent the mean ± sem. Two-way ANOVA with Dunnett *post hoc* test: $^{*}P < 0.05$; $^{**}P < 0.01$; $^{****}P < 0.0001$. $n = 3$ technical replicates.

© 2020 The Authors

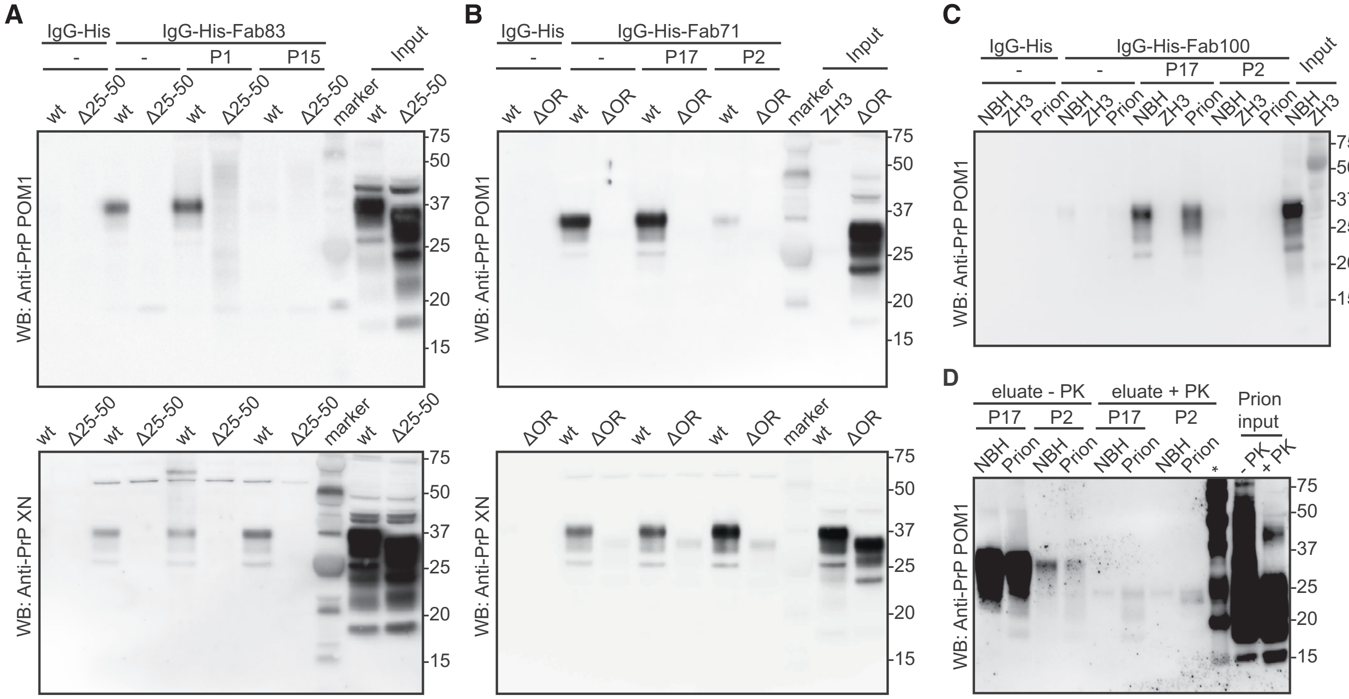

Figure EV4. Validation of the reactivity of the anti-PrP Fabs by immunoprecipitation.

A Fab83 coupled beads immunoprecipitated wtPrP^C from NBH, but not from BH of PrPΔ25–50 mice lacking the respective epitope. WtPrP^C specifically eluted by competition with the Fab83 epitope-targeting peptide P1 (residues 23–34), but not with the unrelated peptide P15 (top panel). Elution with a SDS buffer was used as nonspecific control (lower panel). Control: IgG-His. Molecular sizes are presented in kDa.

B Same as (A), but for Fab71 coupled beads which immunoprecipitated wtPrP^C from NBH, but not from BHs of PrPΔOR mice. WtPrP^C specifically eluted with the epitope-targeting peptide P17 (residues 55–66), but not with the unrelated peptide P2 (top panel).

C IgG-His-Fab100 coupled beads efficiently immunoprecipitated wtPrP^C from non-infectious brain homogenate (NBH) and total wtPrP from brains of prion-infected mice (prion), but not from brains of *Prnp*$^{ZH3/ZH3}$ (ZH3) mice. WtPrP was eluted by competition with the epitope-targeting peptide P17, but not with the unrelated peptide P2. Eluates were visualized by Western blotting. Control: IgG-His. Molecular sizes are presented in kDa.

D Same as (C) for NBH and prion to confirm the specific immunoprecipitation and detection of PrP^{Sc} with the peptide P17 from brains of prion-infected mice. +PK: digested with PK; −PK: non-digested with PK; *: molecular weight markers.

Figure EV5. TR-FRET for the detection of wtPrP^{Sc} in prion-infected CAD5 *Prnp*$^{+/+}$ after treatment with a panel of Fabs.

A TR-FRET for the detection of PrP^{Sc} in prion-infected CAD5 *Prnp*$^{+/+}$ cells after treatment with selected anti-PrP Fabs. Treatment of the cells with Fab71 ($OR_{51\text{-}91}$), but not with Fab3 ($CC1_{23\text{-}50}$) or Fab29 (GD), significantly reduced PrP^{Sc} levels compared to untreated, prion-infected cells.

B Treatment of prion-infected CAD5 *Prnp*$^{+/+}$ cells with Fab100 ($OR_{51\text{-}91}$ binder), but not with Fab83 ($CC1_{23\text{-}50}$ binder), significantly reduced wtPrP^{Sc} levels compared to untreated prion-infected CAD5 *Prnp*$^{+/+}$ cells. The dilution range (3×10^{-5}% to 1% w/v) of prion-infected BH is shown on the right site to indicate the linear range of the assay. Negative controls: NBH and CAD5 *Prnp*$^{-/-}$ cells. Here and in (A), the FRET signal of untreated CAD5 *Prnp*$^{-/-}$ cells was set to zero. The FRET anti-PrP antibody pair POM19-Eu and POM1-APC was used for detection.

Data information: For prion-infected CAD5 *Prnp*$^{+/+}$ cells, the assay was performed in biological triplicates represented as four technical replicates for Fab71, Fab3, Fab100, and Fab83. For CAD5 *Prnp*$^{+/+}$ cells that were either untreated, exposed to NBH, or treated with Fab29, the assay was performed in biological duplicates represented as four technical replicates. For CAD5 *Prnp*$^{-/-}$ cells, one biological replicate was assayed as four technical replicates. $n = 4$–12. Each dilution of prion-infected BH is represented in technical duplicates. Data presented in dot plots represent the mean ± sem. One-way ANOVA and Bonferroni's multiple comparisons were used for statistical analysis; $*P < 0.05$; $**P < 0.01$.

© 2020 The Authors

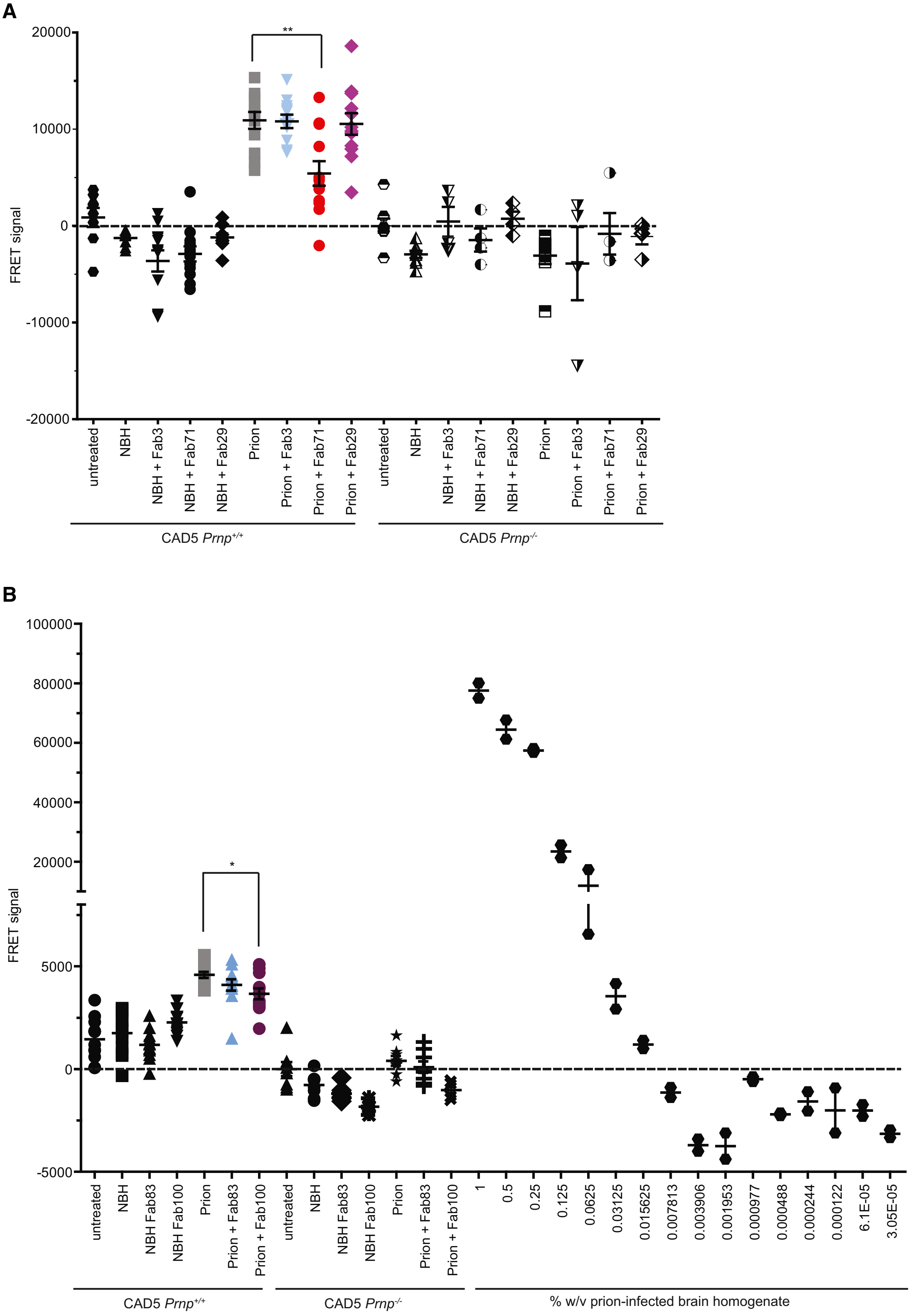
A
**
FRET signal
20000
10000
0
-10000
-20000
untreated
NBH
NBH + Fab3
NBH + Fab71
NBH + Fab29
Prion
Prion + Fab3
Prion + Fab71
Prion + Fab29
untreated
NBH
NBH + Fab3
NBH + Fab71
NBH + Fab29
Prion
Prion + Fab3
Prion + Fab71
Prion + Fab29
CAD5 Prnp+/+
CAD5 Prnp-/-
B
*
FRET signal
100000
80000
60000
40000
20000
5000
0
-5000
untreated
NBH
NBH Fab83
NBH Fab100
Prion
Prion + Fab83
Prion + Fab100
untreated
NBH
NBH Fab83
NBH Fab100
Prion
Prion + Fab83
Prion + Fab100
1
0.5
0.25
0.125
0.0625
0.03125
0.015625
0.007813
0.003906
0.001953
0.000977
0.000488
0.000244
0.000122
6.1E-05
3.05E-05
CAD5 Prnp+/+
CAD5 Prnp-/-
% w/v prion-infected brain homogenate

Figure EV5.
