# Supplementary material for: Protective anti‐prion antibodies in human immunoglobulin repertoires
Source: EMBO Mol Med. 2020 Aug 10;12(9):e12739. doi: 10.15252/emmm.202012739 (PMC7506995; doi:10.15252/emmm.202012739)

Source Data: Uncropped Western blot images

Figure EV4, panel A

- Figure EV4A, Fab83 IP (detection of PrP by POM1)

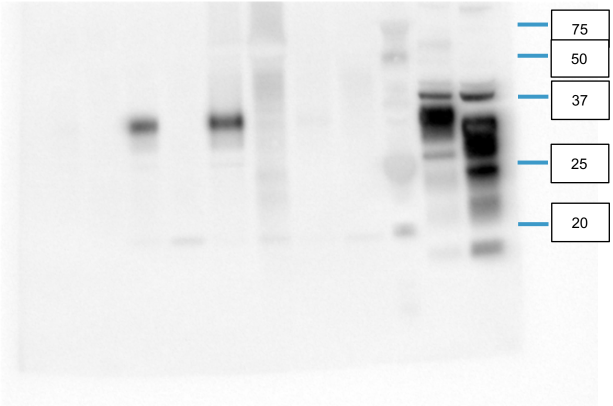

-

- Figure EV4A, Fab83 IP (detection of PrP by anti-PrP XN)

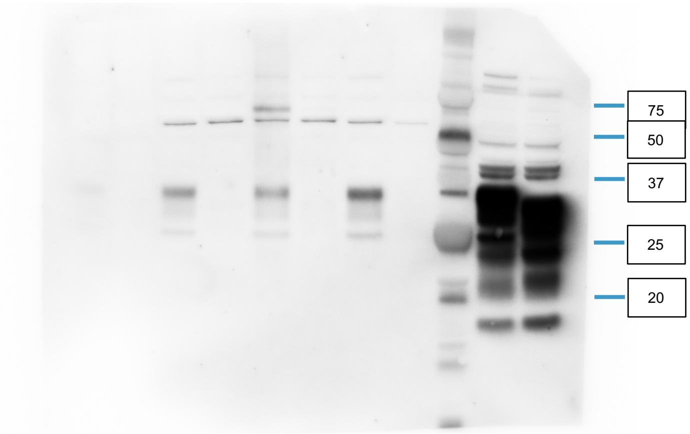

- Figure EV4B, Fab71 IP (detection of PrP by POM1)

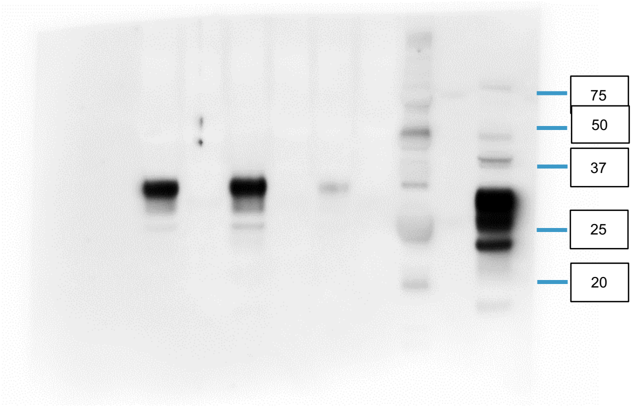

- Figure EV4B, Fab71 IP (detection of PrP by anti-PrP XN)

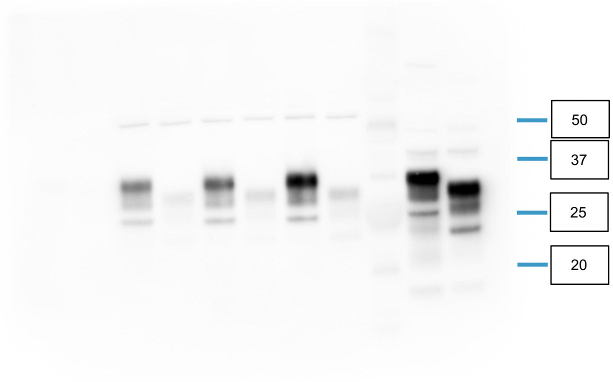

- Figure EV4C, Fab100 IP (detection of PrP by POM1)

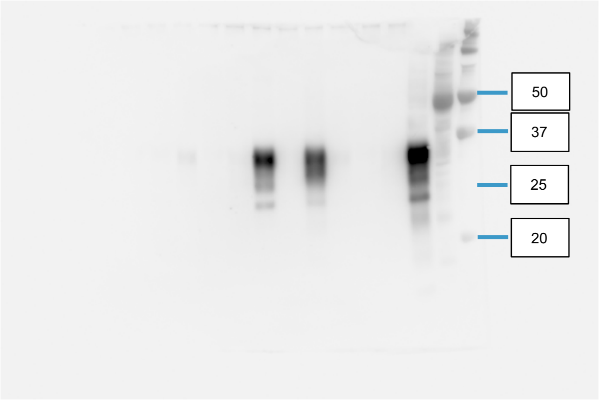

- Figure EV4D Fab100 IP and PK digestion (detection of PrP by POM1)

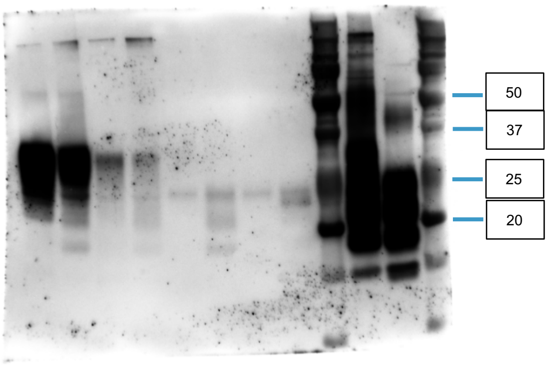

Supplement: Supplementary file 4 — Source Data for Expanded View [file EMMM-12-e12739-s005.zip › EMM-2020-12739-V2-EV_Figure_Source_Data-sd/EMM-2020-12739-V2-Figures_EV_Source_Data-Fig EV4.pdf]
