# Supplementary material for: Protective anti‐prion antibodies in human immunoglobulin repertoires
Source: EMBO Mol Med. 2020 Aug 10;12(9):e12739. doi: 10.15252/emmm.202012739 (PMC7506995; doi:10.15252/emmm.202012739)

Source Data: Uncropped Western blot images

Figure EV3 panel A:

Detection of mouse and human PrP by Fabs: — molecular weight ≈25 kDa

- Figure EV3A, Fab2

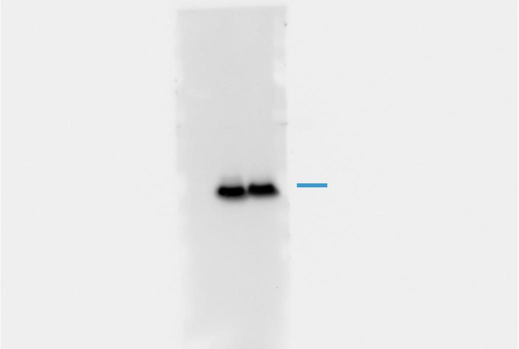

- Figure EV3A, Fab3

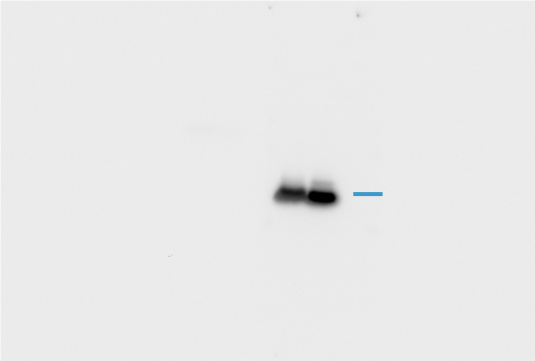

- Figure EV3A, Fab4

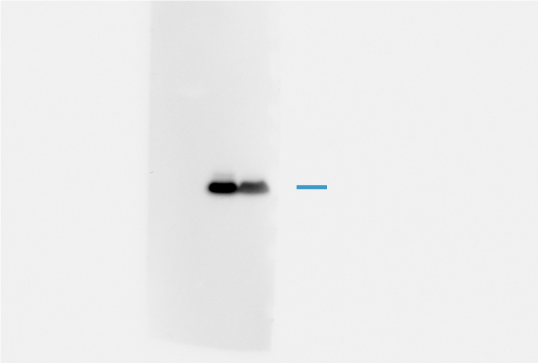

- Figure EV3A, Fab6

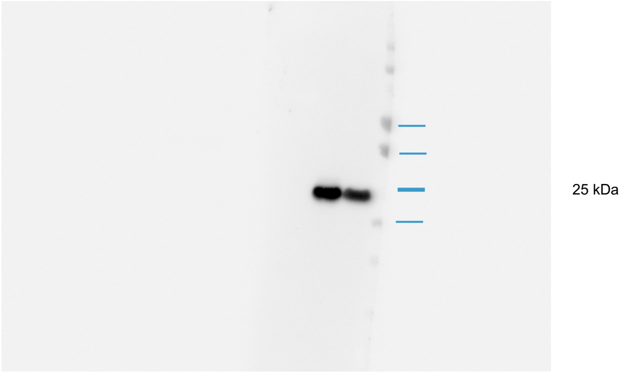

- Figure EV3A, Fab7

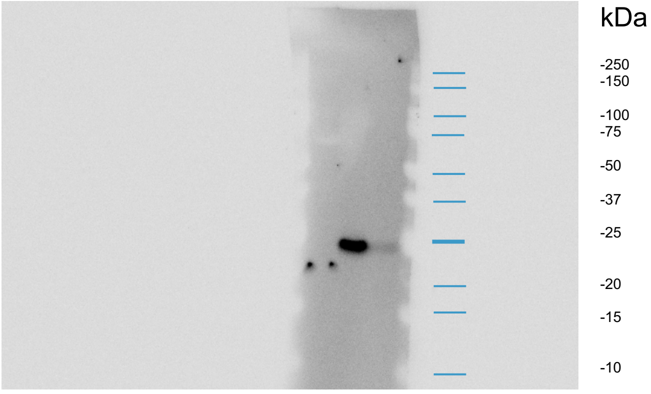

- Figure EV3A, Fab8

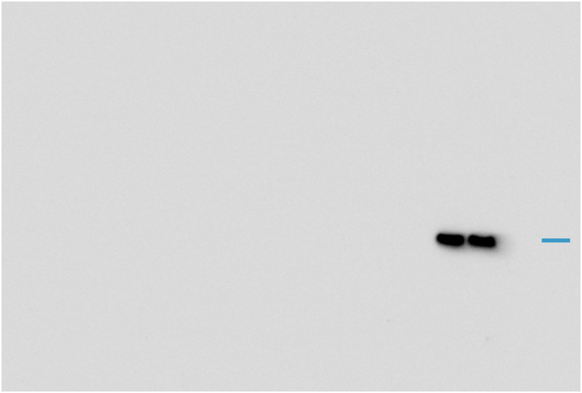

- Figure EV3A, Fab10

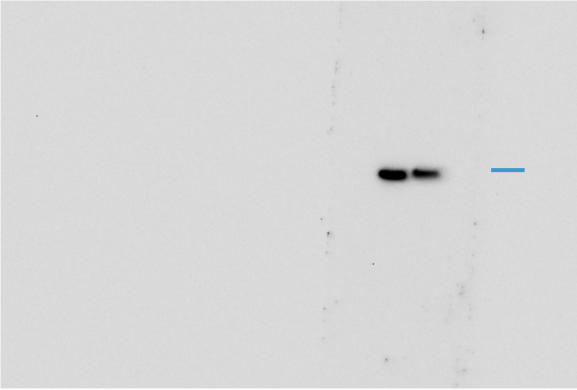

- Figure EV3A, Fab12

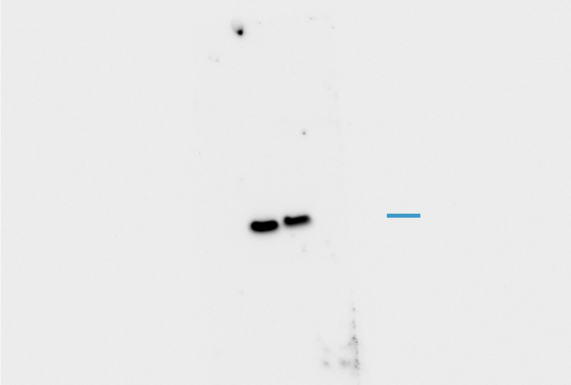

- Figure EV3A, Fab35

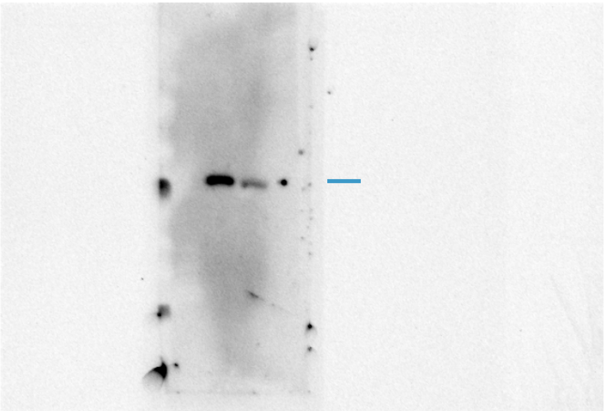

- Figure EV3A, Fab41

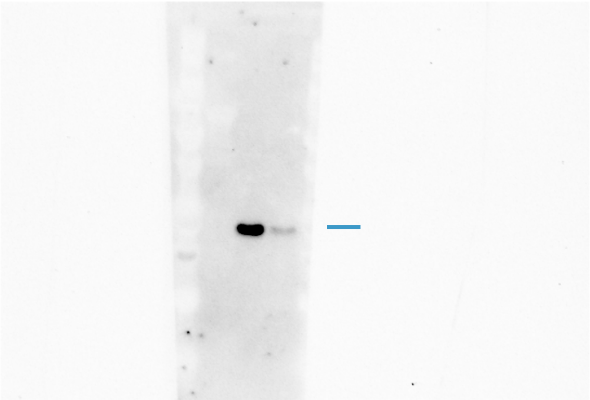

- Figure EV3A, Fab44

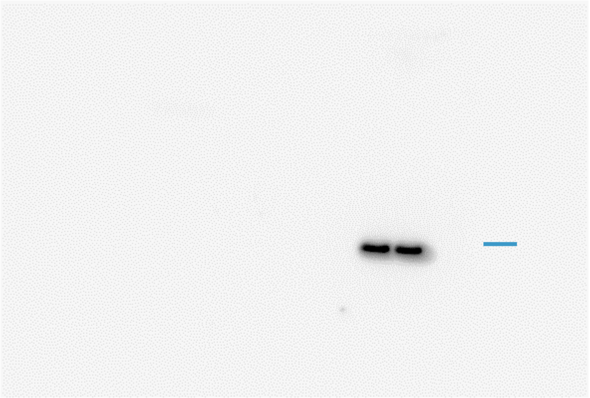

- Figure EV3A, Fab71

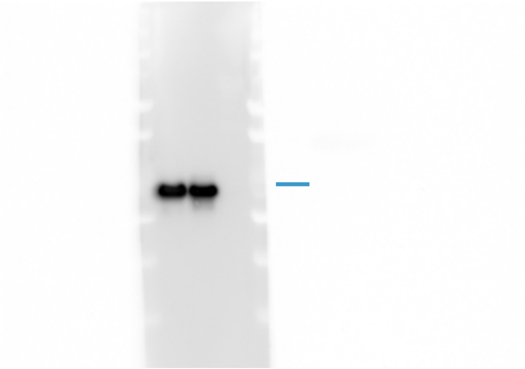

Fab71 molecular weight markers

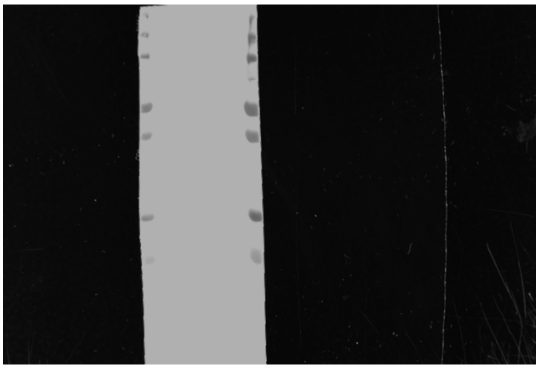

- Figure EV3A, Fab13

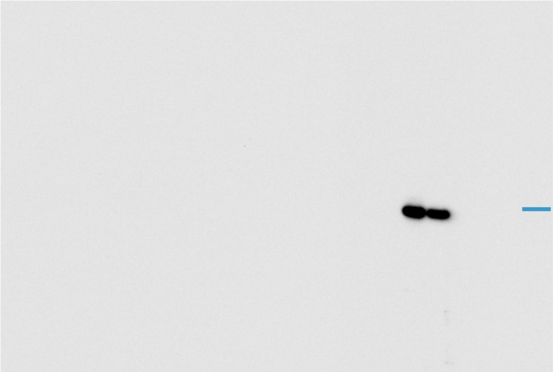

- Figure EV3A, Fab53

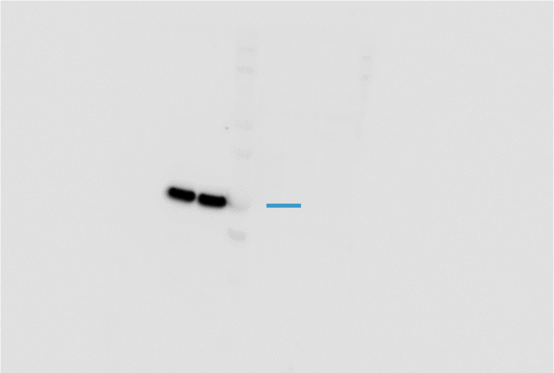

- Figure EV3A, Fab61

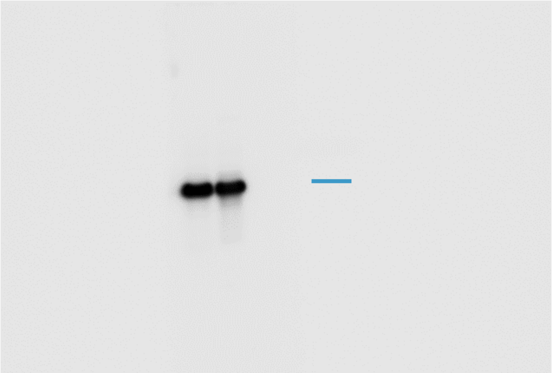

- Figure EV3A, Fab69

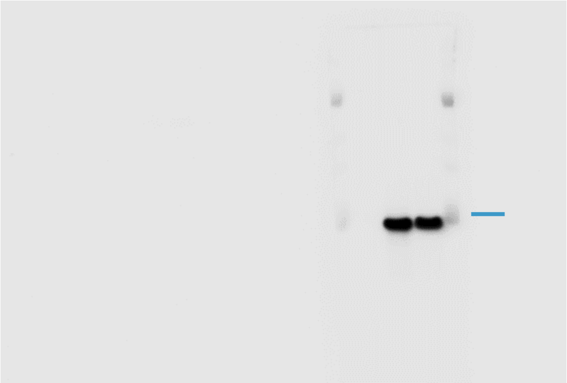

- Figure EV3A, Fab72

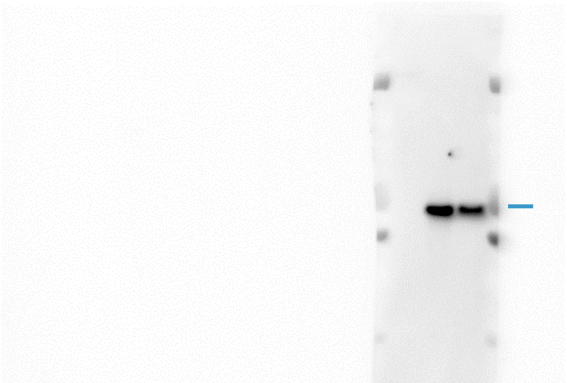

- Figure EV3A, Fab75

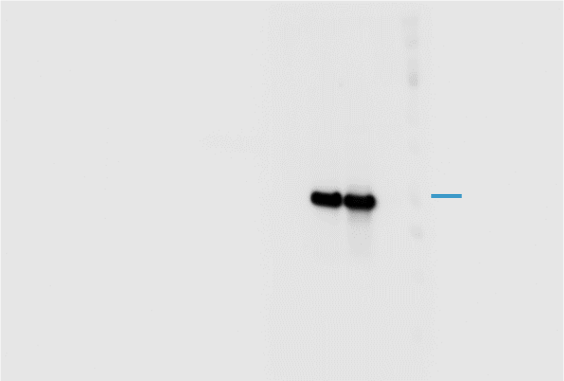

- Figure EV3A, Fab25

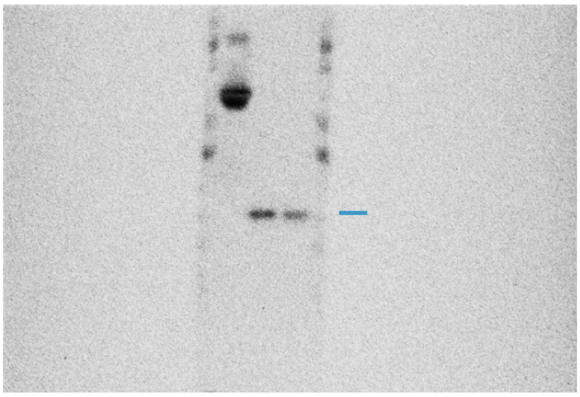

- Figure EV3A, Fab30

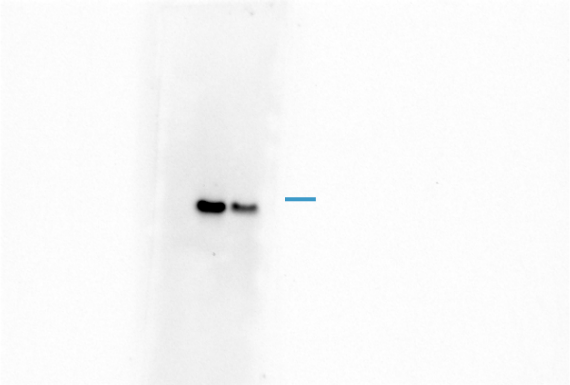

- Figure EV3A, Fab74

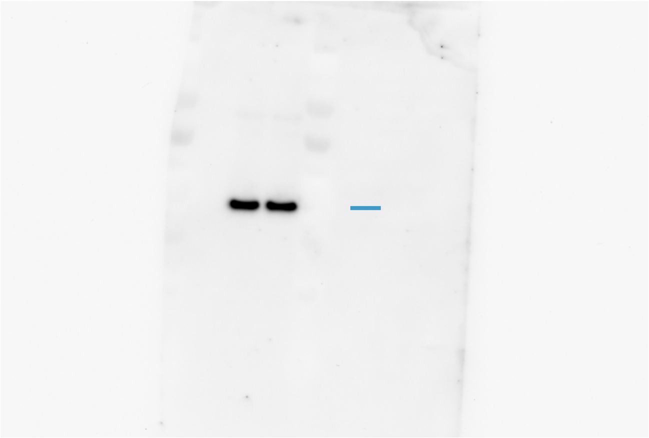

Figure EV3, panel B

Numbers in squares indicate the molecular weight in kDa

- Figure EV3, panel B, Fab83 WB: detection of PrP

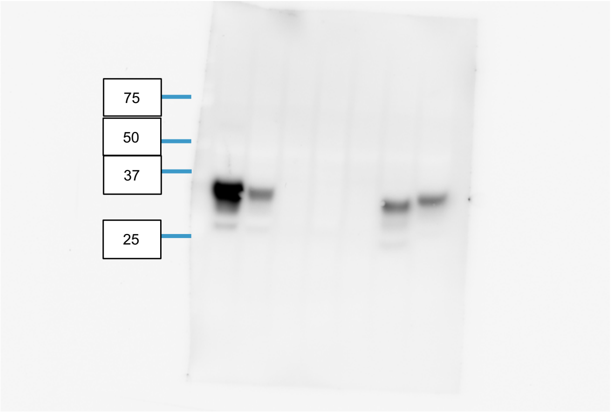

- Figure EV3, panel B, Fab100 WB: detection of PrP

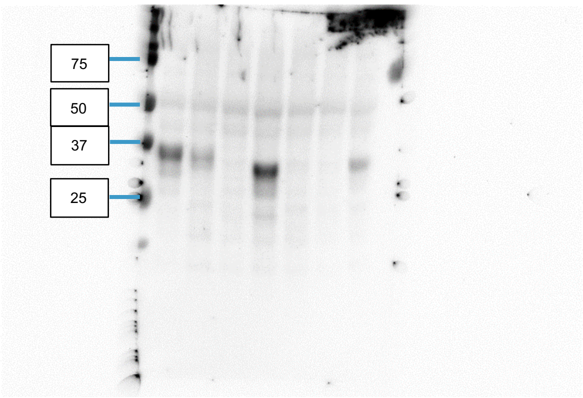

- Figure EV3, panel B, <u>Actin</u> of Fab83 and Fab100 blots

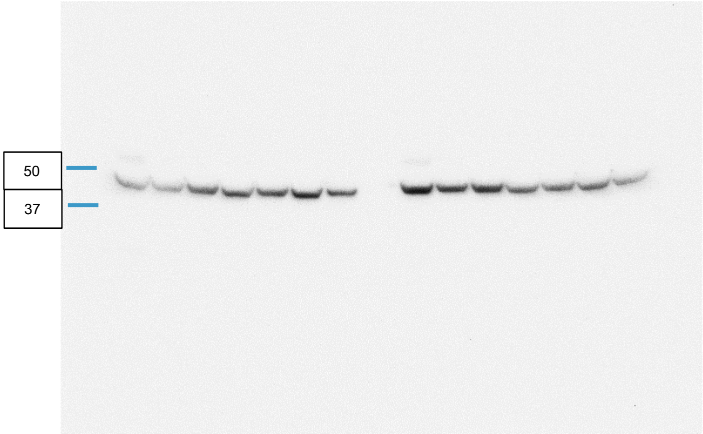

- Figure EV3, panel B, Fab53 WB (detection of PrP)

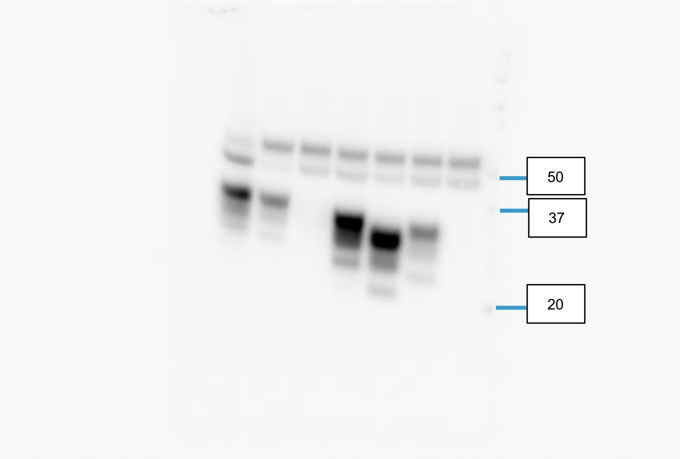

- Figure EV3, panel B, Fab74 WB (detection of PrP)

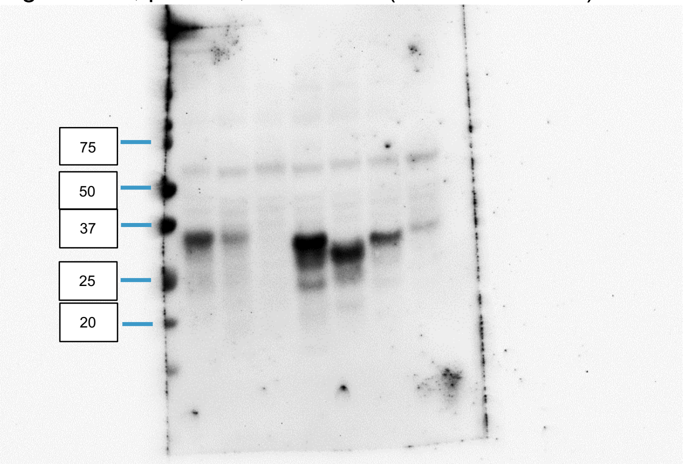

-

- Figure EV3, panel B, Actin of Fab53 and Fab74 blots: the two blots were imaged together

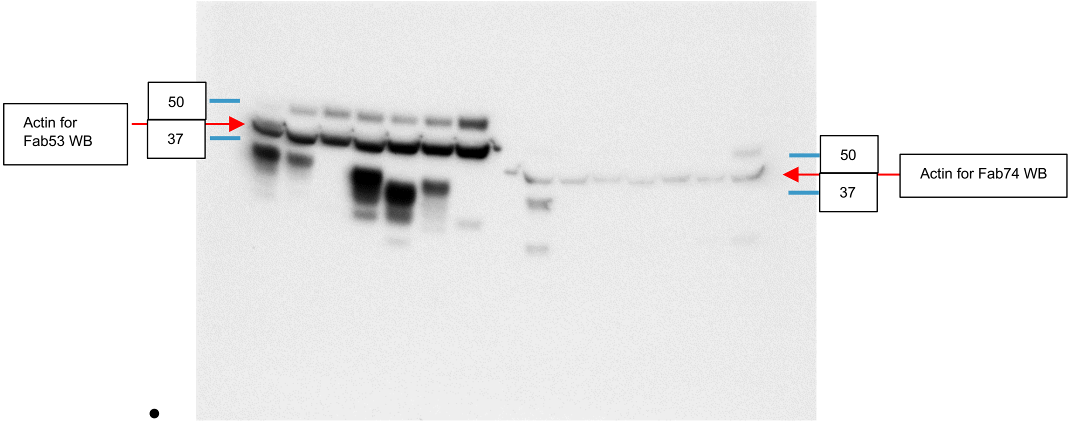

- 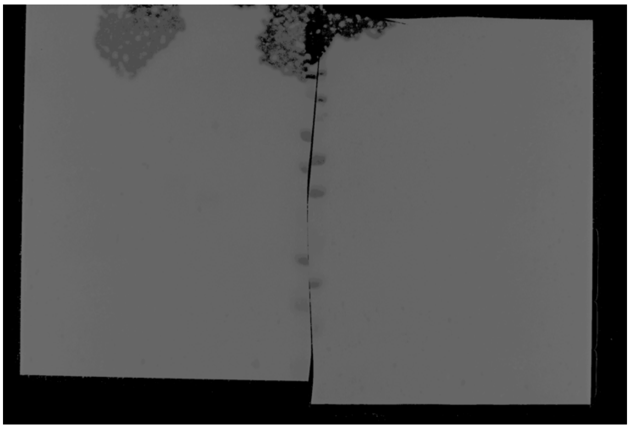

Supplement: Supplementary file 4 — Source Data for Expanded View [file EMMM-12-e12739-s005.zip › EMM-2020-12739-V2-EV_Figure_Source_Data-sd/EMM-2020-12739-V2-Figures_EV_Source_Data-Fig EV3.pdf]
