# Supplementary figures and images for: Protective anti‐prion antibodies in human immunoglobulin repertoires
Source: EMBO Mol Med. 2020 Aug 10;12(9):e12739. doi: 10.15252/emmm.202012739 (PMC7506995; doi:10.15252/emmm.202012739)

## Source Data: Figure 3

### Figure 3, panel D

WB of PrP from COCS lysate

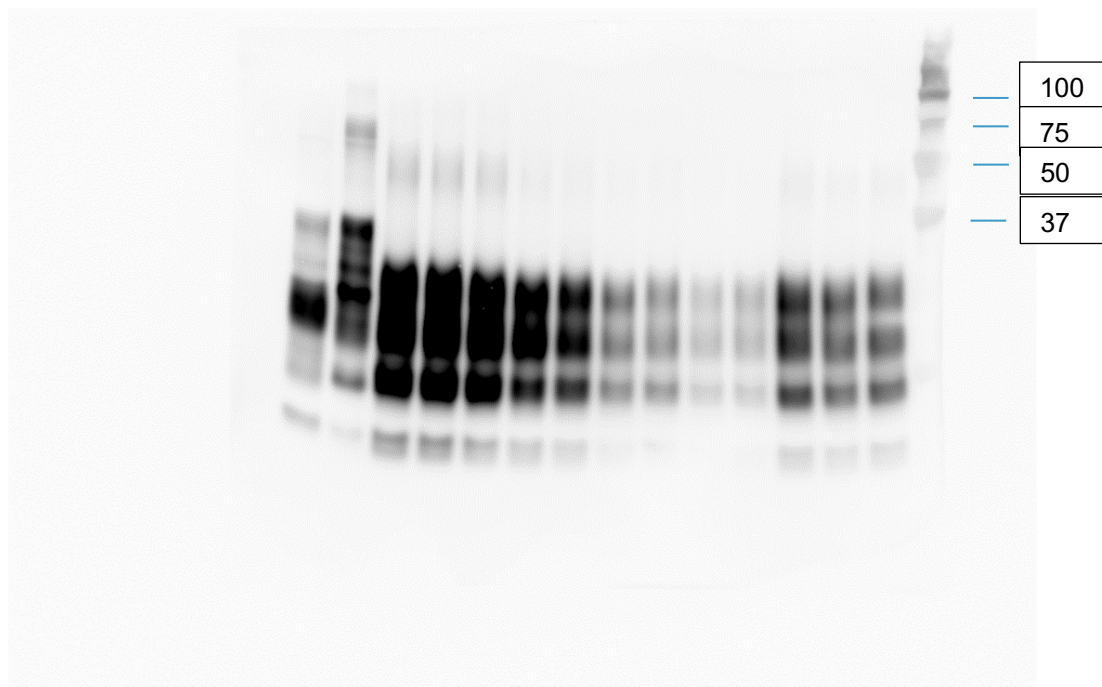

Supplement: Supplementary file 6 — Source Data for Figure 3 [file EMMM-12-e12739-s004.pdf]
